# Purpura Hemorrhagica, Œdema, Anasarca, Etc.*Read before the California State Veterinary Medical Association.

**Published:** 1894-09

**Authors:** H. A. Spencer


### PURPURA HEMORRHAGICA, ŒDEMA, ANASARCA, ETC.*

By Dr. H. A. Spencer.

I have been singularly fortunate in the treatment of this disease, having not lost a case of it in two years, save one that was taken out of my charge on the second day and given over to the tender mercy of a groom. And I have treated a great many, as our locality seems to be a hot-bed for influenza, catarrhal fever, pyogenic fever, or, as it is commonly called, "distemper."

Purpura Hemorrhagica is due to a paralysis of the vaso-motor nerves, which govern the circulation in the smaller blood-vessels

*Read before the California State Veterinary Medical Association.

and capillaries, and also to an altered condition of the blood, the red blood corpuscles being smaller and shrivelled in appearance, as though treated to the action of ammonia. The white corpuscles are increased in number, and there is an excess of the aqueous portions, causing the coagubility of the blood to be greatly lessened.

Purpura usually follows some debilitating disease, as influenza, but has been known when it was the primary disease, due to unsanitary surroundings and poor provinder.

It usually follows a slight attack of catarrhal fever, where the horse has made a rapid recovery, the exciting cause being usually exposure to cold, generally exposure to cold draughts of air on a heated but not necessarily sweaty body, exposure to rain being more apt to cause pneumonia, pleurisy, etc.

Semiology.—The symptoms of this disease are quite variable, but on examination of the tongue you will notice a purple or claret color. Later petecchiæ will appear on the schneiderian membrane, and often in the mouth along the lingual gutter, and on the frænum, then swelling appear around nostrils, on the neck, belly and legs. The tumors are peculiar to this disease ; they are about the size of a hen's egg, well defined, the edges being very abrupt; they pit slightly on pressure, and are but slightly sensitive to the touch. They usually disappear and come again in twelve hours, but not always in the same location as before. These tumors grow rapidly larger, and coalesce, until the legs are twice or three times their normal size; the belly is swollen two to four inches. The line of swelling is very abrupt, as though a ligature had been fastened around ; the animal becomes very stiff and sore from the swelling about the joints. The head sometimes swells to enormous size, especially about the inferior maxillary, making the animal resemble a hippopotamus more than a horse, and inconveniencing him greatly in feeding.

The infusion around the glottis and nostrils sometimes renders breathing difficult. Thus far the temperature is but slightly elevated, if at all ; the pulse is normal, or perhaps only a little weaker; the respiration is not altered, if the œdema does not diminish the caiber of the nostrils or the glottis. The appetite is usually good, and the animal seems in good spirits, but does not move much, on account of the swellings around the articulations.

After three or four days there appears a cereous exudate on the skin over the œdematous parts, that loosens the epidermis, which drys up and cracks. If this exudate is very extensive, there will be ulcerated spots on the skin. The petecchiæ on the schneiderian membrane act as an irritant, and produce a catarrhal discharge. There may or may not be swelling of the lymphatic glands from absorption of the exudate. The temperature now may be slightly elevated, the pulse and respirations accelerated. The appetite is usually good, or may be precarious. The rise in temperature, when it occurs, is caused by the septic poisoning. At this stage there may be sloughing of the skin from the great pressure on it or from poor circulation through it; or gangrene may occur from the same cause. In this case it leaves a large, red, ulcerous sore. Metastasis often occurs. The swelling which has been in the legs and belly suddenly leaves and appears in the head, causing great dyspnœa, and it would seem as though tracheotomy would be necessary, but the animal will almost always die from septicæmia if tracheotomy is performed. Or the swelling may disappear and produce intestinal œdema. This is manifested by great abdominal pain, and usually produces death, or it may terminate in an exhaustive diarrhœa, which is hemorrhagic in nature.

Or it may produce pulmonary œdema In this case there would be marked dyspnœa without swelling of the head This would indicate metastisis into the parenchyma of the lung, showing a dullness on percussion, and ronchas by ascultation. This usually produces death by asphyxia or asthma

Septicæmia is very often the cause of death, especially if there be any sores or wounds on the animal. The symptoms in this case are the usual symptoms of septicæmia, rise in temperature, rigors, hurried breathing, fast, weak, wirie pulse, etc.

Prognosis must always be guarded, as an apparently benign case at first may prove fatal in a very short time, while a very severe case may terminate by resolution in a few days.

TREATMENT:—Medicines must be used to alter the blood, *i. e.*, to produce fibrin factors and more red blood corpuscles, and reduce the watery portions. My line of treatment is usually potassium chloride in half-ounce doses, to produce fibrin factors and reduce the watery portion; iron to stimulate the production of red corpuscles.

Ergot to regulate or give tone to the blood-vessels and quinine as a tonic.

I also use showers of cold water on the œdematous parts to produce a shock to the vaso-motor system controlling the blood-vessels.

